# Towards multimodal visualization of esophageal motility: fusion of manometry, impedance, and videofluoroscopic image sequences

**DOI:** 10.1007/s11548-024-03265-1

**Published:** 2024-10-08

**Authors:** Alexander Geiger, Lukas Bernhard, Florian Gassert, Hubertus Feußner, Dirk Wilhelm, Helmut Friess, Alissa Jell

**Affiliations:** 1 Technical University of Munich, TUM School of Medicine and Health, Klinikum rechts der Isar, Research Group MITI, Munich, Germany; 2 Technical University of Munich, TUM School of Medicine and Health, Klinikum rechts der Isar, Department of Surgery, Munich, Germany; 3 Technical University of Munich, TUM School of Medicine and Health, Klinikum rechts der Isar, Department of Radiology, Munich, Germany

**Keywords:** Multimodal visualization, Medical imaging, Object detection

## Abstract

**Purpose:**

Dysphagia is the inability or difficulty to swallow normally. Standard procedures for diagnosing the exact disease are, among others, X-ray videofluoroscopy, manometry and impedance examinations, usually performed consecutively. In order to gain more insights, ongoing research is aiming to collect these different modalities at the same time, with the goal to present them in a joint visualization. One idea to create a combined view is the projection of the manometry and impedance values onto the right location in the X-ray images. This requires to identify the exact sensor locations in the images.

**Methods:**

This work gives an overview of the challenges associated with the sensor detection task and proposes a robust approach to detect the sensors in X-ray image sequences, ultimately allowing to project the manometry and impedance values onto the right location in the images.

**Results:**

The developed sensor detection approach is evaluated on a total of 14 sequences from different patients, achieving a F1-score of 86.36%. To demonstrate the robustness of the approach, another study is performed by adding different levels of noise to the images, with the performance of our sensor detection method only slightly decreasing in these scenarios. This robust sensor detection provides the basis to accurately project manometry and impedance values onto the images, allowing to create a multimodal visualization of the swallow process. The resulting visualizations are evaluated qualitatively by domain experts, indicating a great benefit of this proposed fused visualization approach.

**Conclusion:**

Using our preprocessing and sensor detection method, we show that the sensor detection task can be successfully approached with high accuracy. This allows to create a novel, multimodal visualization of esophageal motility, helping to provide more insights into swallow disorders of patients.

## Introduction

**Fig. 1:**
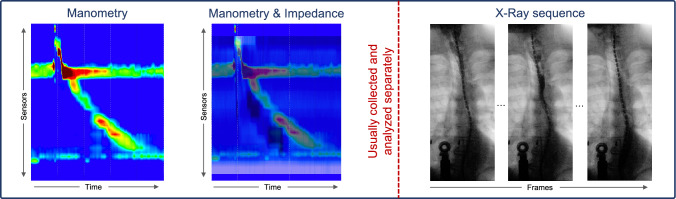
Examples of the three modalities manometry, impedance (overlaid on manometry), and X-ray images (videofluoroscopy) during a single swallowing act. Usually, the modalities are separately collected and analyzed, while in the present work we collect the modalities simultaneously and visualize the different modalities in a combined fashion

Dysphagia is the inability or difficulty to swallow normally. Studies show that 1 of 6 adults in the US reported experiencing difficulty in swallowing [1]. The symptoms range from rather light problems up to being unable to swallow even saliva. The standard procedures for diagnosing the disease are a combination of esophageal manometry and impedance, functional assessments, and videofluoroscopy [2]. Usually, the different examinations are performed consecutively, but recent research is also heading toward performing these different types of examinations at the same time [3]. The goal of the simultaneous capturing of different modalities is to create an encompassing visualization of the esophageal motility during the swallowing act of a patient.

Figure 1 shows examples of collected manometry and impedance values, as well as a corresponding X-ray sequence. The manometry and impedance values are visualized using Clouse Plots [4] to better show the values over time. The dynamic X-ray sequence shows the movement of the bolus (water with contrast agent) and gives a good indication of the anatomy of the patient as well as the general esophageal motility during a swallowing act.

## Motivation

**Fig. 2:**
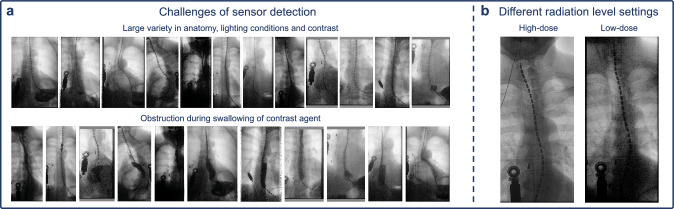
Examples showing the challenges of the sensor detection in the X-ray images (**a**), as well as a comparison between a high-dose and a low-dose X-ray image of the same patient (**b**)

The reason to create a multimodal visualization of the esophageal motility of a patient is to provide a more intuitive way for clinicians to analyze the collected data. One idea to combine them into a single visualization is the projection of manometry and impedance values onto the right location in the X-ray images. Jell et al. [3] describe in more detail why such a fusion of modalities is desired for the examination of esophageal disorders. They also provide a template matching-based approach to detect the sensors. However, their sensor detection method was developed for high-dose X-ray fluoroscopy which typically results in sharper and higher contrast images. For low-dose X-ray sequences, which result in lower quality images and which are the norm for examinations of patients in order to reduce harmful X-ray exposure, the previous method is not sufficient (see Fig. 2b for an example of the quality difference between a high-dose and a low-dose image). In this work, we aim to provide a solution that is applicable for low-resolution X-ray images.

## Related work

In the field of medicine, object detection is widely used to detect certain structures in images. For example, Gierlinger et al. [5] use a region growing method to segment elongated structures like blood vessels in breast MRI. Another use case is the detection of stents or catheters in fluoroscopic images. In this area, multiple approaches were developed to tackle this task. For example, Ma et al. [6] use a framework of blob detection, shape-constrained searching and model-based detection, while Chabi et al. [7] use an adaptive thresholding and subsequent classifier to detect stents and catheter markers in X-ray fluoroscopy images. Reiml et al. [8] use the radial symmetry transform to detect stent markers before removing outliers from the detected results and fitting a line or ellipse that is overlaid on the image, while Bismuth et al. [9] developed an approach that combines the detection and tracking of a stent as well as the contrast enhancement of the images. While these approaches were mostly based on traditional detection methods, more recent work focuses on using machine learning-based approaches to detect catheters in X-ray images. In this domain, Yi et al. [10] provide an overview of existing algorithms for the assessment of catheters on radiographs. For example, Yi et al. [11] train a scale-recurrent segmentation network on synthetic data, while Henderson et al. [12] use a convolutional neural network to classify multiple catheter types in X-ray images and Dai et al. [13] use supervised attention U-Net architecture to segment catheters in MR images. Moreover, Lee et al. [14] and Yu et al. [15] propose machine learning-based methods aiming to detect peripherally inserted central catheters in chest X-ray images.

When it comes to the combination of manometry values with other image-based modalities, Davidson et al. [16] overlay manometric values on a 3D visualization of a colon. In order to analyze esophageal motility, Jell et al. [3] used a template matching approach to detect manometry sensors in esophageal X-ray sequences, focusing on high-dose images. In this work, we extend this approach to low-dose X-ray images and additionally include impedance values as an additional visual component.

## Challenges

The presented sensor detection task in low-resolution images provides a couple of challenges that make the accurate detection of the sensors difficult.

*Varying lighting conditions, anatomy, catheter curve* Due to the different anatomy of every patient, as well as radiologist-dependent X-ray settings (custom windowing, varying sampling rate, etc.), the X-ray sequences and the course of the catheter can differ a lot between patients, requiring a robust method that is able to cover all those cases.

*Not all sensors visible* It is often the case that not every sensor along the catheter is visible in the X-ray image. If one or more sensors are not visible due to obstruction/low contrast, their location still has to be inferred as precise as possible, otherwise the subsequent projection of the manometry and impedance values would not be possible. When it comes to the accuracy of the sensor detection, we observed that the predicted sensor location should deviate at most 5 pixels from the real location to have a perfect match, while a deviation of up to 30 pixels is still acceptable in order for the projections to still look correct and realistic on top of the underlying image. Figure 2a shows examples of the variety in anatomy, lighting conditions and contrast, as well as how the view of the sensors can be obstructed due to contrast agent.

*Required correct detection of first or last sensor* Due to the rather narrow imaging of the patients’ esophagus, usually not all 36 sensors along the catheter can be seen. For detecting the catheter, however, the X-ray sequences must deliberately be taken in a way that either the top or the bottom of the catheter is visible, as otherwise there would be no automatic way of identifying which of the 36 sensors are shown in the image. This also means that the first (or, respectively, last) sensor has to be reliably detected in the image, as it serves as the only reference of the sensor numbers that are shown.

## Methods

In order to overcome the presented challenges, we propose a sensor detection method that is able to detect the sensors accurately in all frames, even if the visibility of some sensors is obstructed (e.g., by contrast agent or the high-density mandibular region). Since only a limited number of patient data was available at the time of the development, we decided to use a template matching-based approach that is not requiring large, labeled data sets compared to machine learning-based methods.

**Fig. 3:**
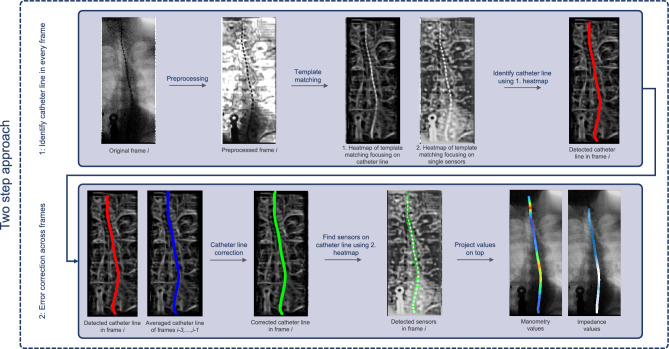
Our two-step approach to detect the sensors and project the manometry and impedance values onto the right location

### Setup

The X-ray images have a dimension of $1024 \times 1024$ pixels, and any gray and black borders are automatically cropped out at the beginning of the process. They are sampled at a rate of 8–15 frames per second. Depending on the swallow duration and sample rate, the resulting sequences consist of around 40–180 frames in total. Manometry and impedance values are collected using a state-of-the-art high resolution catheter (50 samples per second) with 36 circular manometry sensors distributed at a distance of 1 cm along the catheter. In the X-ray images, the typical number of visible sensors in a frame is around 30, the distance between two sensors is typically around 35–40 pixels, with a sensor being around 25–30 pixels long (depending on the X-ray settings used for a given patient). The distance between two sensors can also vary within an image in case the esophagus is bending the catheter; however, no overlap of two sensors has been observed in the investigated cases so far. The 15 impedance values are measured between every second sensor, starting from the fourth sensor. The X-ray images, as well as manometry/impedance, are collected at the same time and therefore reflect the same esophageal events.

### Preprocessing

Preprocessing of the images is the first step in order to provide the best conditions for later sensor detection. We found the best option to be a combination of noise removal and contrast enhancement and implemented this by first applying a Gaussian blur filter to remove noise from the image, and then applying the local contrast-enhancement algorithm CLAHE [17].

### Sensor detection

After preprocessing, the actual sensor detection takes place. In the following, we present our approach which is based on a template recognition algorithm using the OpenCV-based template matching function. Due to the challenges listed before, solely applying this technique was not yielding good results in our initial experiments, as only the most clearly visible sensors were detected. Therefore, in order to detect all sensors accurately, we developed a two-step approach, which can be seen in Fig. 3.

**Algorithm 1:**
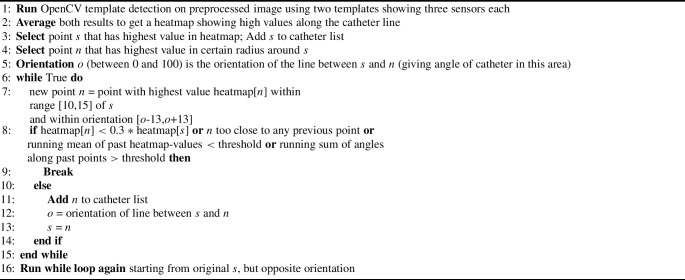
Detect catheter line in image

After preprocessing, the initial step involves detecting the catheter line in each frame using OpenCV template matching. Two templates, featuring three sensors each (one with a larger sensor size and one with a smaller size for increased robustness), are employed. The resulting heatmap, displaying high values along the catheter line, allows precise location determination of the catheter. Algorithm 1 outlines the detailed catheter line detection process.

**Fig. 4:**
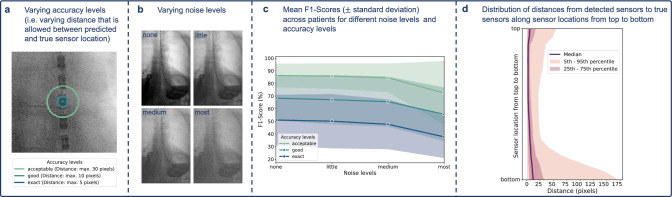
Details of the sensor detection evaluation. **a** Showing the three levels of accuracy (i.e., distances of a detected sensor to the true sensor that are considered to be a correct detection); **b** showing the four varying noise levels; **c** showing the F1-scores for the three accuracy levels and four noise levels, indicating only small performance decrease with more noise, while achieving the best results with the lowest accuracy level, as expected; **d** showing the distribution of distances from the detected sensors to the true sensors along the sensor location, ranging from top to bottom. As observed also in the qualitative evaluations, greater distances are more often observed at lower sensors, arguably due to worse image quality in the lower image areas

Due to performance variations across frames, especially when contrast agent obstructs the catheter, an error correction step is introduced. Using catheter locations from the previous three frames, the algorithm corrects the current frame’s location if it deviates too much from the previous ones. This iterative approach corrects deviations in all subsequent frames.

With the catheter line identified in each frame, the next step involves localizing individual sensors along the catheter. A second heatmap, created by template matching with two templates (this time featuring a single sensor each), aids in this process. Algorithm 2 provides a detailed description of the sensor localization procedure.

**Algorithm 2:**
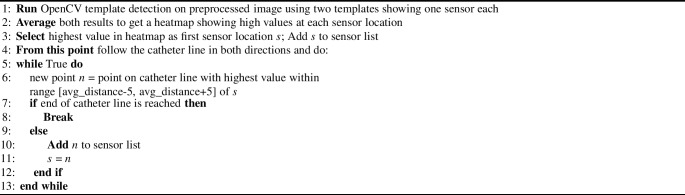
Detect single sensors along catheter line

### Projection of manometry and impedance values onto sensor locations

As a last step, the corresponding manometry and impedance values are projected on top of the correct sensors in each frame. Both the X-ray fluoroscopy and the manometry/impedance data were collected at the same time; therefore, for each X-ray frame the associated manometry and impedance values can be projected on top of the respective sensor location. For visualization purposes, the projected values are color coded similar to the Clouse Plots mentioned in section “Introduction” . The manometry and impedance values between the single sensor locations are interpolated in order to get a continuous colored line.

## Results

To assess our method, synchronized videofluoroscopy and manometry/impedance data were collected during the swallow process from 14 patients, approved by the ethics committee of the University Hospital rechts der Isar, Munich, Germany. We then both quantitatively and qualitatively evaluated the obtained results together with medical experts. Despite the simplicity of our approach, the experiments show that it is effectively addressing the challenges outlined in section “Challenges” .

**Table 1:** Sensor detection results with different noise levels and accuracy levels

Noise level	Accuracy level	Precision (%)	Recall (%)	F1-score (%)
None	Exact	$50.64 \pm 20.81$	$52.07 \pm 19.58$	$51.07 \pm 19.90$
	Good	$67.65 \pm 19.38$	$69.74 \pm 16.84$	$68.35 \pm 17.71$
	Acceptable	$85.23 \pm 12.15$	$88.31 \pm 8.46$	$86.36 \pm 9.30$
Little	Exact	$49.71 \pm 22.53$	$51.21 \pm 20.82$	$50.13 \pm 21.43$
	Good	$66.40 \pm 21.55$	$68.78 \pm 18.06$	$67.15 \pm 19.48$
	Acceptable	$84.38 \pm 13.62$	$88.40 \pm 9.00$	$85.81 \pm 10.03$
Medium	Exact	$47.65 \pm 20.88$	$44.88 \pm 22.09$	$47.66 \pm 19.60$
	Good	$65.42 \pm 22.11$	$61.91 \pm 24.65$	$65.59 \pm 19.77$
	Acceptable	$83.83 \pm 14.85$	$80.17 \pm 24.27$	$84.53 \pm 11.29$
Most	Exact	$38.51 \pm 17.89$	$29.71 \pm 21.38$	$37.72 \pm 16.73$
	Good	$56.70 \pm 22.61$	$43.81 \pm 29.71$	$55.59 \pm 21.18$
	Acceptable	$73.71 \pm 26.62$	$57..22 \pm 37.80$	$72.45 \pm 25.23$

We report the averaged metrics across all patients with the respective standard deviation (±)

**Fig. 5:**
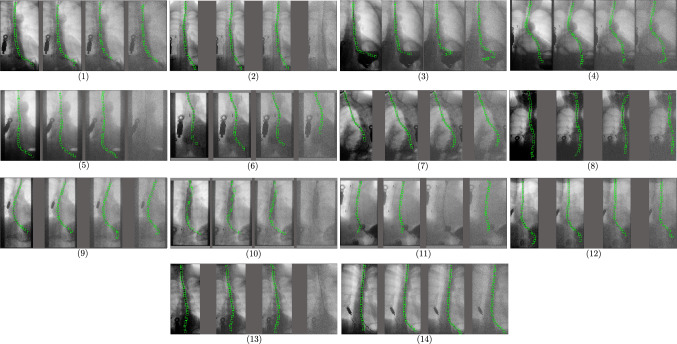
Sensor detection performance when adding different levels of Gaussian noise to the fluoroscopy images of each patient. Four levels per patient, from no noise (left-most images) to most noise (right-most images)

**Fig. 6:**
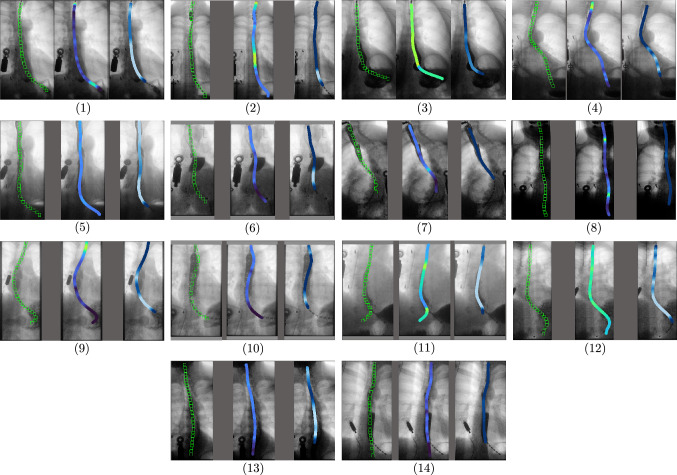
Output of our algorithm showing sensor location and projected manometry and impedance for 14 different patients during the swallowing act. In most images, the contrast agent can be seen, obstructing multiple sensors, while the sensor detection is still able to infer the correct sensor locations, allowing to accurately project the manometry and impedance values onto the correct location in the image

### Sensor detection

Overall, the catheter’s general course is correctly identified for all of the patients, highlighting the method’s robustness in discovering the correct object in the X-ray images. To quantify the sensor detection accuracy, the true sensor locations in a total of 515 frames (on average 37 frames per patient and around 30 visible sensors per frame) were meticulously annotated. The sensor detection performance is then measured using the metrics: precision, recall and F1-score. We provide the scores for three levels of accuracy, i.e., “exact”, “good”, and “acceptable”. A detection is considered correct if the identified sensor is within a 5-pixel radius of the true sensor position at the “exact” accuracy level, within a 10-pixel radius at the “good” accuracy level, and within a 30-pixel radius at the “acceptable” accuracy level. While the highest accuracy level essentially represents an exact detection, the distance of at most 30 pixels in the lowest accuracy level is still considered to be acceptable for our given task, verified by qualitative evaluations (see also Fig. 4a for an example of the three levels of accuracy on a sample sensor).

Using these accuracy levels, our evaluation shows a mean F1-score across all patients of 51.07% for the “exact” accuracy level, 68.35% for the “good” level, and 86.36% for the “acceptable” level. This means that the locations of roughly half of the sensors are perfectly detected, while the locations of most of the remaining sensors are detected at least at an acceptable level.

In order to further evaluate the robustness of the approach, an additional study was conducted where different levels of Gaussian noise were added to the images before applying the sensor detection method. Figure 4b shows an example of the four levels of noise tested in this scenario. As can be seen in Fig. 4c, the detection performance is only slightly decreasing with additional noise, still achieving an F1-score in the acceptable accuracy level and highest noise level of 72.45%. All results, including precision, recall, and F1-score, can be found in Table 1.

Confirming the quantitative results, a qualitative evaluation of the results indicates a consistently good sensor detection as well. It can be observed that the sensor detection is working especially well in the center area of the images, while the sensors in the lower parts are sometimes not detected as accurately. This trend can also be seen in Fig. 4d, which shows the distribution of distances from the detected sensors to the true sensors along the catheter curve from top to bottom. While the median distance is not varying much from top to bottom, it still can be observed that the number of cases with high distances to the correct sensor increases for the lower parts of the sensor catheter. We argue that this is due to the generally worse contrast and visibility of the sensors in the lower area, mainly due to accumulating contrast agent and occluding anatomical parts in this lower area of the esophagus.

Figure 5 shows examples of the sensor detection obtained in the evaluation, including different levels of noise. Similar to the quantitative results, it can be observed that light noise does not influence the sensor detection in most cases, and even with strong noise the sensors can be detected for the majority of patients, demonstrating that the sensor detection approach is robust even in cases of unusually low image quality.

### Projection of manometry and impedance values

The results demonstrate the effectiveness of our method to detect the sensors in the videofluoroscopy images, which is the basis of the subsequent multimodal visualization. This is achieved by projecting manometry and impedance values onto the correct locations in the images.

Figure 6 depicts the sensor detection results as well as the resulting multimodal visualization, fusing videofluoroscopy with manometry and impedance data. The images show the most challenging middle phase of a swallowing act for each of the patients. This phase mostly involves visible contrast agent that is obstructing parts of the catheter. We observe a successful sensor detection during this phase through our error correction across frames, consequently allowing to accurately project the manometry and impedance values throughout the complete swallow process.

Qualitative evaluations by upper gastrointestinal (GI) motility experts affirmed the significance of these multimodal visualizations, emphasizing the valuable new insights into esophageal motility offered by combining X-ray frames with corresponding manometry and impedance values.

## Conclusion

We outlined the challenges in detecting sensors in esophageal X-ray sequences and introduced a robust template matching-based sensor detection method. Our method accurately detects sensors for a majority of evaluated patients, achieving a F1-score of 86.36% at detecting the sensors. Subsequently, this enables a precise projection of manometry and impedance values onto X-ray images, that allows to create a novel fused visualization of the three modalities. Testing the approach on a total of 14 patients, our combined visualization offers valuable insights for better examining esophageal disorders, as confirmed by upper GI motility experts.

While the current results show a generally accurate sensor detection, certain cases (particularly in the lower part of the catheter) exhibit detection issues, requiring future efforts to enhance accuracy in this area.

Additionally, our ongoing data collection aims to expand the dataset in order to allow an evaluation across a larger patient dataset. The increased dataset will also facilitate the exploration of alternative sensor detection methods. Initially relying on template matching due to limited data, our ongoing research now includes developing a machine learning-based sensor detection approach, allowing for a comparative analysis to the existing approach.

Furthermore, a crucial future focus involves clinically evaluating the multimodal visualization approach. Further collaborations with upper GI motility experts will provide insights into the combined visualization’s benefits and any additional requirements. Additionally, other modalities should be considered in the future as well, aiming to present clinicians all necessary information in an optimal visual format.
